# One Health approach to *Coxiella burnetii* in Brazilian indigenous communities

**DOI:** 10.1038/s41598-024-60850-9

**Published:** 2024-05-02

**Authors:** João Henrique Farinhas, Danilo Alves de França, Maria Carolina Serpa, Leandro Meneguelli Biondo, Fernando Rodrigo Doline, Rogério Giuffrida, Vamilton Alvares Santarém, Andrea Pires dos Santos, Marcelo B. Labruna, Louise Bach Kmetiuk, Alexander Welker Biondo

**Affiliations:** 1 Graduate College of Cell and Molecular Biology, Federal University of Paraná (UFPR), Curitiba, PR 80035-050 Brazil; 2 Department of Animal Production and Preventive Veterinary Medicine, School of Veterinary Medicine and Animals Science, São Paulo State University, Botucatu, SP 18618-681 Brazil; 3 Department of Preventive Veterinary Medicine and Animal Health, School of Veterinary Medicine and Animal Science, University of São Paulo, São Paulo, SP 05508-270 Brazil; 4 National Institute of the Atlantic Forest (INMA), Brazilian Ministry of Science, Technology, and Innovation, Santa Teresa, ES 29650-000 Brazil; 5 Graduate College in Animal Sciences, University of Western São Paulo (UNOESTE), Presidente Prudente, , São Paulo Brazil; 6 Department of Comparative Pathobiology, Purdue University, West Lafayette, IN 47907 USA

**Keywords:** Diseases, Risk factors

## Abstract

Indigenous health has posted complex challenges worldwide, particularly due to historical economic, territorial, social and environmental processes, which may lead to emergence and reemergence of pathogens. In addition to few *Coxiella burnetii* serosurveys in vulnerable populations, especially in developing tropical countries, no comprehensive One Health approach has focused on human-animal infection along with potential environmental determinants. Accordingly, this study aimed to assess the seroprevalence of anti-*C. burnetii* antibodies in indigenous populations and their dogs from 10 indigenous communities distributed in southern and southeastern Brazil, along with the correspondent healthcare professionals. In overall, 8/893 (0.90%; 95% CI 0.45–1.76) indigenous and 1/406 (0.25%) dog samples were seropositive, with 7/343 (2.04%) individuals the 1/144 (0.69%) dog from the Ocoy community, located in the city of São Miguel do Iguaçu, bordering Argentina at south, and far 10 km at west from Paraguay. All 84 healthcare professionals tested seronegative.

## Introduction

Indigenous health has posted complex challenges worldwide and particularly in Brazil due to historical economic, territorial, social and environmental processes which may lead to emergence and reemergence of pathogens^1^. An overall 1,693,535 indigenous persons, accounting for 0.83% of the nationwide population, was recently estimated by the 2022 Brazilian Census and included 305 ethnic groups speaking more than 274 languages^2^. As these populations mostly live in remote areas, under basic infrastructure and struggling to maintain their primitive ancestral culture, native indigenous communities have been considered among the highest social vulnerability in Brazil^1^. In such a scenario, the close human-animal-environmental interaction may also impact determinants of indigenous health, particularly with expansion of livestock production and clandestine mining, pushing these populations even closer to endemic and novel pathogens, under absence of basic health conditions^1^.

Q fever has been described as a worldwide underreported and underdiagnosed zoonosis, mostly due to initial asymptomatic infection leading to nonspecific symptoms, making difficulty prompt and proper diagnosis. However, disease may progress and trigger cardiac and neurological problems, particularly in patients with persistent infection^3^.

The Q fever causative agent, *Coxiella burnetii*, exposure has been reported in 85/887 (9.6%) indigenous Orang Asli population of Malaysia^4^, and in 11/267 (4.1%) indigenous persons of James Bay native communities in Canada^3^. Although with not fully established epidemiology, these studies have associated *Coxiella burnetii* seropositivity with geographical and demographic factors, and with wildlife contact^4^. In addition, Australian aboriginal dogs were 2.8-fold more likely infected than purebred, domestic and shelter dogs^5^. Finally, a single study in South America has found no exposure out of 73 individuals tested in 2014 and 2015 at the indigenous Halite-Paresi community, located in the central-western Brazil^6^.

Although considered underdiagnosed and with little occurrence data in Latin America^3^, *C. burnetii* infection has reportedly affected cattle and small ruminant populations in southern and southeastern Brazil, causing animal abortions and exposing human populations up to 30 km away from the infected farms^7–9^. Although febrile illness has been commonly associated with several other diseases, *C. burnetii* may persist in the body, causing chronic complications such as endocarditis, hepatitis, and meningitis^10,11^. As underestimated disease, *C. burnetii* impact may be even higher in indigenous communities, with no confirmed cases or serological exposure evidence to date in Brazil.

In addition to few *C. burnetii* serosurveys in vulnerable populations, particularly in developing tropical countries, no comprehensive One Health approach to date has surveyed the *C. burnetii* infection in indigenous populations, characterized by their close relationship with natural forest areas and wildlife animals. Accordingly, this study aimed to assess the seroprevalence of anti-*C. burnetii* antibodies in indigenous persons, their dogs and healthcare professionals from 10 indigenous communities distributed in southern and southeastern Brazil.

## Results

**Figure 1:**
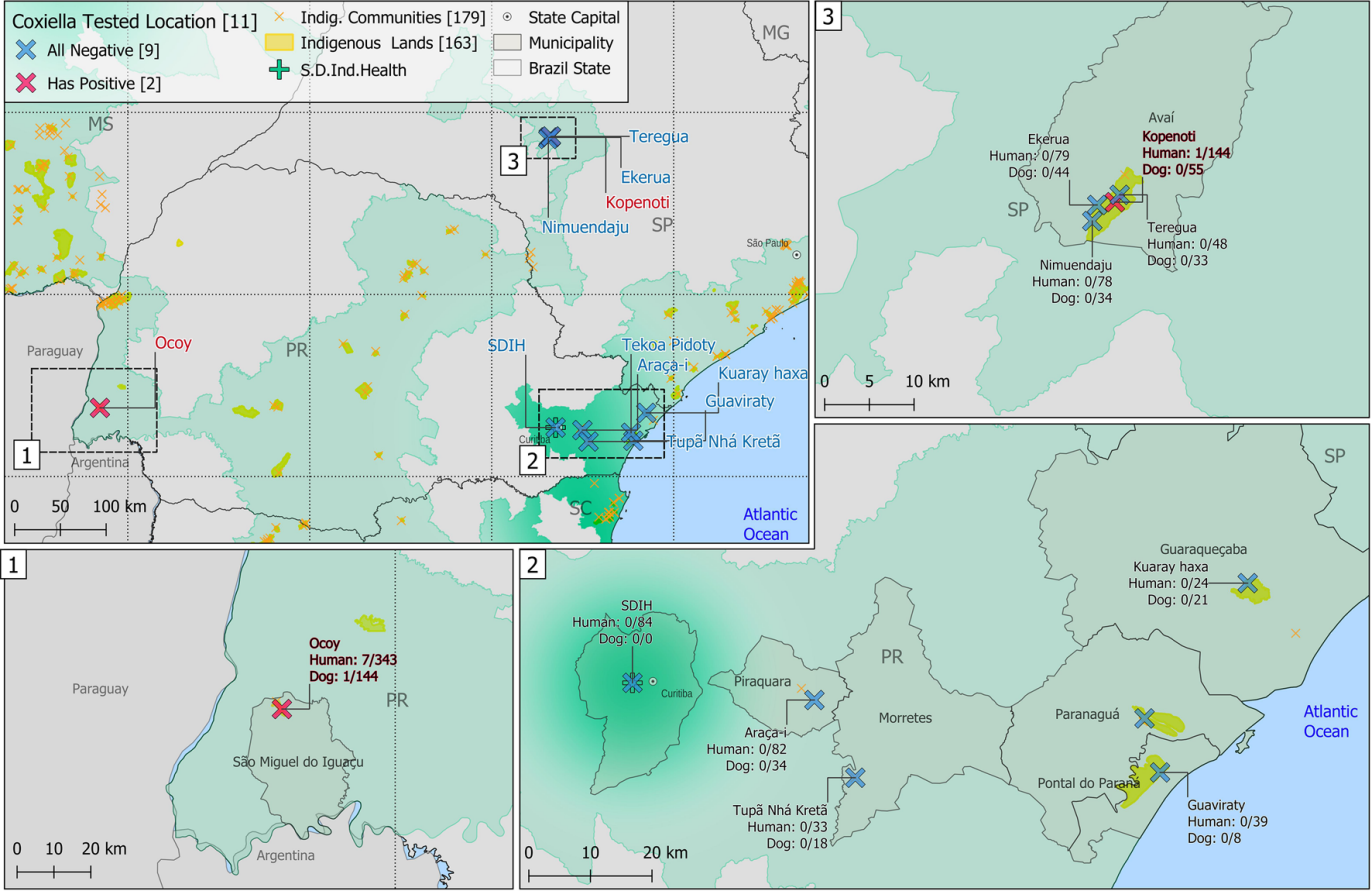
Sampling locations and distribution of *C. burnetti* exposure in indigenous communities of Paraná and São Paulo states, size distance invariance hypothesis (SDIH) of national catching areas. All components of the map were open source and open data-direct link to the source of icons and symbols used: https://github.com/qgis/QGIS/tree/master/images/svg; direct link of the boundaries from the officially public data of the Brazilian government, used as background base layer: https://geoftp.ibge.gov.br/cartas_e_mapas/bases_cartograficas_continuas/bc250/versao2021/post_gis/bc250_2021_11_18.zip; public indigenous areas and SDIH areas for shading and location: https://geoserver.funai.gov.br/geoserver/web/. Map produced by the authors using QGIS 3.18.

A total of 893 human and 406 dog serum samples were obtained from the 10 indigenous communities, including 343 individuals and 144 dogs at Ocoy community, 144 and 55 at Kopenoty, 82 and 34 at Araça’í, 79 and 44 at Ekeruá, 78 and 34 at Nimuendajú, 48 and 33 at Tereguá, 39 and 8 at Guaviraty, 33 and 18 at Tupã Nhe’e Kretã, 24 and 21 at Kuaray Haxa, and 23 and 15 at Pidoty. In addition, the 84 healthcare professionals were sampled during the incursions to the indigenous communities (Fig. 1).

Overall, 8/893 (0.90%; 95% CI 0.45–1.76) indigenous and 1/406 (0.25%) dog samples presented anti-*C. burnetii* antibodies, with 7/343 (2.04%) individuals and the 1/144 (0.69%) dog from the Ocoy community, located in the city of São Miguel do Iguaçu, bordering Argentina at south, and far 10 km at west from Paraguay. All 84 healthcare professionals tested negative. Among the positive human samples, six presented 1:128 titers, one 1:64 and one 1:256, while the dog titers were 1:128. Separating by community, seven of the positive human samples and the positive dog samples were from the Ocoy community, Paraná. Human seropositivity in the Ocoy community was 2.04% (7/343, 95% CI 1.00–4.15) and dog seropositivity was 0.70% (1/144, 95% CI 0.12–3.83). The other positive human sample was from Kopenoty community, São Paulo state. Table 1 shows the results of the analysis of risk factors associated with *C. burnetii* positivity in indigenous communities. It was observed a statistical significance between the seropositivity for *C. brunetti* and the habit of hunting.

**Table 1:** Associated risk factors for anti-*Coxiella brunetii* antibodies (IgG) in the indigenous population in south (Paraná state) and southeast (São Paulo state) Brazil (N = 690) by univariate analysis (Exact Fisher Test).

	Results			
	Positive (%)	Negative (%)	Odds Ratio (95% CI)	*p* value
	n = 7	n = 683		
Gender				0.706
Female	3 (42.9)	379 (55.5)	1.0 (Reference)	
Male	4 (57.1)	304 (44.5)	1.64 (0.34–8.96)	
Age (years)				1.0
Up to 15	2 (28.6)	190 (28.1)	1.0 (Reference)	
16–23	1 (14.3)	151 (22.4)	0.67 (0.02–8.35)	
24–37	2 (28.6)	166 (24.6)	1.14 (0.12–11.1)	
> 37	2 (28.6)	168 (24.9)	1.13 (0.12–11.0)	
Hunting habit				0.049
No	3 (42.9)	532 (77.9)	1.0 (Reference)	
Yes	4 (57.1)	151 (22.1)	4.63 (0.96–25.3)	

## Discussion

This is the first report of anti-*C. burnetii* antibodies in indigenous communities of Brazil. Ocoy was the most seropositive indigenous community, presenting 7/8 human and the single dog cases. As specific characteristics, Ocoy community was the closest to bordering countries, with frequent international crossing to visit other indigenous communities located nearby in Argentina and Paraguay. Also, this community was the most inserted into wildness, historically transferred from their original area (which was submerged) and living at the artificial lakeshore of the Itaipu Company, effective in 1982 and the biggest electric powerplant dam in Brazil since, currently the third worldwide in electricity production. The other only seropositive human sample was detected at the Kopenoty community, São Paulo state, likely exposed outside the indigenous area.

Vulnerable populations worldwide have been reportedly more susceptible to infections in general, due to factors such as social inequality, housing conditions, lack of basic sanitation and basic hygiene habits^9^. In Brazil, descendants of former black slaves have shown 44/200 (22.0%) seropositivity to anti-*C. burnetii* antibodies, indicating a higher risk of exposure of such vulnerable population when compared to general population. Nonetheless, geographical isolated location, cultural habits and other associated factors may have prevented Brazilian indigenous populations from their exposure to *C. burnetii*, as only 8/893 (0.90%) seropositive samples were found herein. In addition, no previous detection was made out of 73 indigenous individuals in the Amazon of central-western Brazil, serosurveyed in 2 consecutive years^6^.

The Ocoy community may present unique geographical and social characteristics among the indigenous communities surveyed herein. As already mentioned, the original Ocoy indigenous area was confiscated and later submerged by the Itaipu electric power plant dam, a binational company assembled on the Paraná river at Iguassu city, between Brazil and Paraguay, nearby the Argentinian border^12^. The Iguassu city was created to house the 20,000 employees at the time, currently the second biggest Brazilian city throughout the 15,735 km (9778 miles) terrestrial border, with 285,500 habitants^12^. Thus, the growth and significant changes may have influenced the wildlife relationship and predispose circulation of *Coxiella* and other infectious agents in such formerly isolated indigenous communities.

Despite the indigenous habits obtained herein were not statistically associated as risk factors for Q fever, low seropositivity have impaired proper analysis. Moreover, the habits of the Ocoy community were similar to those of other surveyed communities. Nonetheless, wildlife contact during hunting and trapping was statistically associated with leptospirosis but not with Q fever due to < 5% seroprevalence in 267 James Bay residents^4^, while < 1% prevalence was observed in 917 blood samples from Nunavik residents^13^, both near Quebec, Canada. Finally, two indigenous Orang Asli communities closer to forest areas in Malaysia were significantly more likely seropositive than the other surveyed villages, with an overall 9.6% (85/887) seroprevalence^4^.

In contrast, human populations living outside indigenous communities of southern and southeastern Brazil have shown higher *C. burnetii* seroprevalence, including 129/604 (21.5%) urban febrile individuals in São Paulo^9^ and 21/437 (4.8%) in Minas Gerais states^7^, and 44/200 (22.0%) rural descendants of former black slaves in the Paraná state^14^. The absence of exposure in indigenous communities within the same areas may be explained by the considerable distance between such populations and cattle and sheep farms, livestock species frequently associated with *C. burnetii* infection and environmental contamination in Brazil^8,15,16^. Likewise, absence in the Kuaray Haxa, Pidoty and Guaviraty indigenous communities, all located at environmentally preserved seashore and oceanic island areas, have also indicated that livestock distance may be an associated protective factor for *C. burnetii* exposure.

Although Q fever transmission may occur by contact with dog vaginal fluids after giving birth, the role of dogs in the human cycle remains unclear^6^. The only case reported to date has described a Q fever outbreak affecting three family members with pneumonia, following exposure to an infected parturient dog and death of all puppies^17^. In addition, a single study comparing dogs from native communities with domestic and shelter dogs has shown that Aboriginal dogs in Australia were 2.8 times more likely infected than other dog groups^5^. Such pattern was not observed herein in dogs of Brazilian indigenous communities, with only 1/144 (0.69%) seropositive dog. Thus, further studies should be conducted to fully establish the zoonotic potential and associated risk factors of Q fever between dogs and humans. Noteworthy, the study herein was also the first assessment in indigenous dog populations to the *C. burnetii* infection in the Americas.

As pre-Columbian Brazilian indigenous communities (estimated of 1,000 different ethnicities with 5–13 million habitants in 1500) have never domesticated any native wildlife species, exotic domestic animals brought from Portuguese and other European settlers including companion, livestock, and pest species had a disastrous impact in the original indigenous lifestyle^18^. The presence of domestic dogs (and cats) has been historically increased nationwide in almost all Brazilian indigenous communities, even in communities deeply isolated into the Amazon region as the Yanomamis, currently aggravated by pet abandonment in surrounding areas (by illegal settlers, miners, and hunters) associated to uncontrolled pet reproduction, and lack of veterinarian assistance including vaccination, deworming and tick control^19^. As a serious historical consequence, dogs have played an important role in several zoonotic diseases in Brazilian indigenous communities including toxocariasis, toxoplasmosis, tungiasis, strongyloidiasis, rabies, leptospirosis, leishmaniasis, Chagas disease and rickettsiosis^19^. Thus, the gap in Q fever occurrence and impact in these communities has led to the pioneer study herein.

Herein, although the odds ratio estimation was not statistically significant considering the 95% CI (lower limit was close to 1.0), the seropositivity for *C. brunetii* was associated with the habit of hunting. A relevant risk of exposure to tick-borne pathogens, including *C. brunetii*, has been considered for hunters during hunting activities^20^. Therefore, it is possible to argue that the indigenous population evaluated herein are prone to be exposed to tick bite and becoming infected by *C. burnetii* while hunting.

As limitations, although no cattle, horses, sheep, or swine were allowed (or observed) in the indigenous communities, the present study has not mapped or surveyed livestock animals in the surroundings areas as associated risk factors. Particularly in the Ocoy indigenous community, livestock raising and production in nearby farms may be a potential source for the *C. burnetii* exposure found herein, and should be further investigated.

Finally, future studies should be conducted in nearby Paraguayan and Argentinean indigenous communities, to pinpoint and fully establish the source of *C. burnetii* infection, and potential spreading by terrestrial international crossing of indigenous individuals. In addition, future pneumonia patients from the Ocoy community should be surveyed for Q fever as rule out diagnosis, as a guideline model for surveillance and prevention activities in Brazil and other endemic countries.

This is the first report of anti-*C. burnetii* antibodies in indigenous communities of Brazil, mostly at the Ocoy indigenous community, with frequent international border crossing to visit other indigenous communities located nearby in Argentina and Paraguay, serving as an alert for international spreading of disease. Future surveys should be conducted in Paraguayan and Argentinian indigenous communities cross the border, to fully establish such infection risk and transmission.

## Material and methods

### Ethics statement

This study was approved by the Human Health Ethics Committee at the Brazilian Ministry of Health (protocol 52.039.021.9.0000.0102). Before participating in the present study, informed consent was obtained from all participants and/or their legal guardians. All procedures were performed in accordance with relevant guidelines and regulations.

### Indigenous communities

The serosurvey herein was carried out in the ethnic groups of Guarani, Terena and Kaingang from 10 different indigenous communities, located in the Paraná state (six communities) at southern and São Paulo state (four communities) at southeastern Brazil. All six indigenous communities in the Paraná state were situated within the Atlantic Forest biome at seashore (3), countryside (2), and far-west (1) areas, while all four communities in São Paulo state were situated nearby each other, within the Cerrado biome.

The six indigenous communities of Paraná state included Tupã Nhe’e Kretã, Araça’í, Pidoty, Kuaray Haxa, Guaviraty, and Ocoy. These communities were characterized by living on natural resources for survival such as fishing and agriculture, in close contact with wildlife, and with poor basic hygiene infrastructure. The Kuaray Haxa, Pidoty, and Guaviraty communities were located on seashore and oceanic islands, where biodiversity and traditional finishing culture have been well-preserved, while Tupã Nhe’e Kretã and Araça’í were situated in preserved areas of the metropolitan area of Curitiba, state capital and the eighth biggest Brazilian city with 1.8 million habitants. Finally, the far-west Ocoy community was marked by traditional indigenous countryside culture, high people movements through Paraguayan and Argentinean borders, and majority of individuals speaking only Guarani (and not the official Portuguese language).

The four indigenous communities of São Paulo state were located nearby and included Kopenoty, Tereguá, Ekeruá and Nimuendajú. These communities were less traditional in indigenous culture than those in the Paraná state and had adequate basic sanitation, living mostly by agriculture as the main family activity income. Many indigenous people worked outside the communities, in nearby towns or on rural properties.

### Healthcare professionals

**Figure 2:**
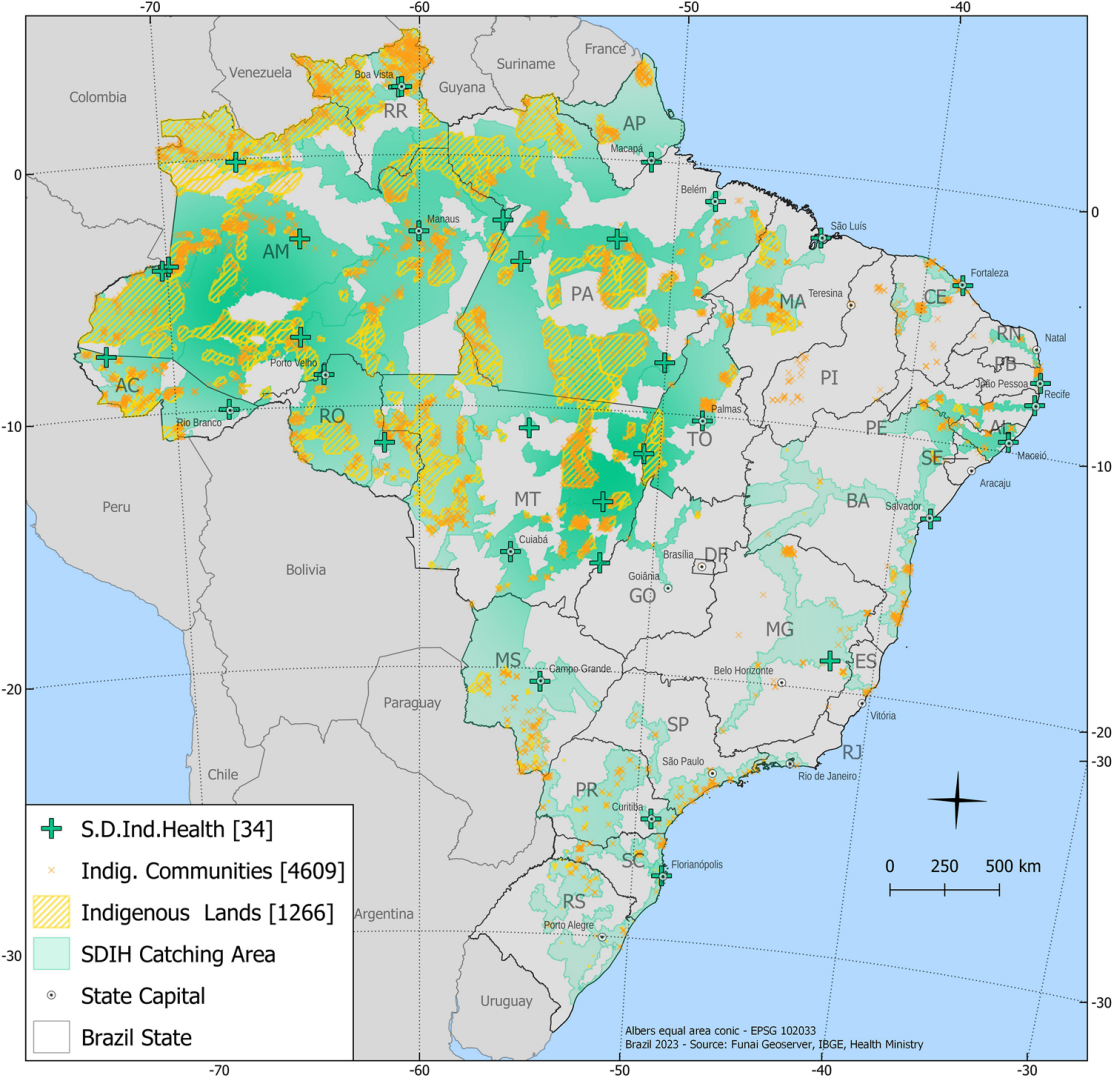
The 34 Special District of Indigenous Health (SDIH) centers and correspondent areas within the geographical map of Brazil, including the SDIH—Seashore South (number 13). All components of the map were open source and open data—direct link to the source of icons and symbols used: https://github.com/qgis/QGIS/tree/master/images/svg; direct link of the boundaries from the officially public data of the Brazilian government, used as background base layer: https://geoftp.ibge.gov.br/cartas_e_mapas/bases_cartograficas_continuas/bc250/versao2021/post_gis/bc250_2021_11_18.zip; public indigenous areas and SDIH areas for shading and location: https://geoserver.funai.gov.br/geoserver/web/. Map produced by the authors using QGIS 3.18.

Healthcare professionals working in the indigenous communities through the National Unified Health System (SUS) were also invited to voluntarily participate in the serosurvey. Blood samples were collected at the headquarters—Special Department of Indigenous Health (SDIH) Seashore South (Fig. 2). The health professionals were grouped according to their level of contact with the population (high: doctors, nurses, drivers, and teachers; medium: multidisciplinary teams; low: administrators), their role in the communities and the frequency of their visits.

### Study design and sample collection

After signing an individual voluntary consent, human blood samples were collected from indigenous people and health professionals by cephalic venipuncture performed by certified nurses, and dog blood samples by jugular venipuncture performed by certified veterinarians. All blood samples were centrifuged at 800*g* for 5 min, serum placed in cryotubes and kept at − 20 °C until processing. Epidemiological data was collected using individual questionnaires, mostly helped by an indigenous translator, to obtain information including on age, gender, and hunting habits. All indigenous people living in these locations were included in the study, with no age limit. Dog owners also answered epidemiological questionnaires with information on their dogs including age, gender, race, and hunting habits.

### Serological analysis

For human and dog serodiagnosis, anti-*C. burnetii* antibodies were detected by immunofluorescence with a previously established protocol^21^, using *C. burnetii* strain At12-infected Vero cells as antigen. Human-specific and dog-specific IgG fluorescein isothiocyanate (FITC) antibodies were used. Positive and negative controls were obtained from human and dog serum samples, previously tested in the herein laboratory, with positive and negative titers of 1:16. Phosphate buffered saline (PBS) with a pH of 7.2 was used to create sequential dilutions at ratios of 1:16, 1:64, 1;128, 1:256, 1:512, 1:1024, and 1:4096. Samples were considered positive when antibody titers were equal or greater than 64. For seropositive samples, final titers were determined as the last dilution in which ≥ 50% of bacterial fluorescence remained apparent. As this in-house test was not designed to differentiate antibody phases, the study herein has not identified acute from chronic seropositive patients. However, the aim of the present study was to assess serological prevalence of anti-C. burnetii antibodies in healthy vulnerable populations^21^. In addition, such diagnostic tests have proven to be as effective at detecting anti-phase II antibodies as anti-phase I antibodies, which could lead to the possibility of detecting chronic patients in further studies^21^.

### Statistical analysis

The association between anti-*C. burnetii* antibodies (IgG) and risk factors (gender, age, and hunting habit of indigenous) was evaluated by the Fisher exact test (univariate analysis) due to the low number of seropositive cases (7/690). The odds ratio (OR) was calculated with 95% confidence intervals (95% CI) by median biased estimation. All statistical analysis was conducted in R software v. 4.2.3 (R core team). A *P* value of < 0.05 was considered significant.

## Data Availability

The datasets generated during and/or analyzed during the current study are available from the corresponding author on reasonable request.
